# Healthcare Resource Utilization Among Patients With Agitation in Alzheimer Dementia

**DOI:** 10.36469/001c.124455

**Published:** 2024-10-29

**Authors:** Christie Teigland, Zulkarnain Pulungan, David Bruhn, Steve Hwang

**Affiliations:** 1 Inovalon Insights, Bowie, Maryland, USA; 2 Otsuka America Pharmaceutical, Inc., Princeton, New Jersey, USA

**Keywords:** Agitation, Alzheimer’s disease, dementia, Medicare claims, healthcare resource utilization, burden of disease

## Abstract

**Background:** Agitation in Alzheimer dementia is common, but the associated healthcare burden remains unclear. **Objective:** This retrospective analysis evaluated baseline characteristics, healthcare resource utilization, and costs among patients with agitation in Alzheimer dementia and those without agitation in Alzheimer dementia. **Methods:** Medicare beneficiaries from 100% of the Medicare Fee-for-Service claims database (2009-2016) with 2 or more claims 30 or more days apart for both Alzheimer’s disease and dementia and continuous enrollment with medical/pharmacy coverage for 6 months before and 12 months after the index diagnosis were included. Patients with agitation in Alzheimer dementia were identified by 2 or more claims 14 or more days apart using *International Classification of Diseases-9-CM/-10-CM* codes based on the provisional International Psychogeriatric Association agitation definition. Patients with severe psychiatric disorders were excluded. Two cohorts of patients (with and without agitation) were then defined, and patient characteristics, healthcare resource utilization, and costs were compared in a descriptive exploratory analysis. **Results:** Of 2 684 704 Fee-for-Service patients with Alzheimer dementia, 769 141 met all inclusion criteria; among these, 281 042 (36.5%) had agitation. The mean age in patients with and without agitation in Alzheimer dementia was 83 years. Most patients in both groups were female, but the proportion of males was slightly higher in the agitation in Alzheimer dementia group (30.3% vs 28.2%, respectively). Patients with agitation in Alzheimer dementia were more likely than those without agitation in Alzheimer dementia to have lower socioeconomic status (dual eligibility for Medicaid, 45.0% vs 41.7%, respectively) or be disabled (10.5% vs 9.4%). Overall, healthcare costs were higher in the agitation in Alzheimer dementia population compared with those without agitation in Alzheimer dementia (mean cost PPPY, 32 322and30 121, respectively), with the largest differences observed in inpatient and post-acute care costs. **Conclusions:** These exploratory findings underscore the substantial economic burden of agitation in Alzheimer dementia and highlight the need for treatment options for the agitation in Alzheimer dementia population to improve associated health outcomes.

## INTRODUCTION

Alzheimer’s disease (AD) is the most common cause of dementia in the United States, affecting 6.7 million individuals aged 65 years or older in 2023 and projected to impact 13.8 million people by 2060 without the intervention of therapies that slow or halt disease progression or cure AD.^1^ Agitation, which is characterized by abnormal motor activity, restlessness, verbal and/or physical aggressiveness, and emotional distress, occurs in approximately 30% to 76% of patients with dementia associated with AD.^2^ The presence of agitation in Alzheimer dementia has been associated with greater caregiver stress, increased risk of institutionalization, greater healthcare use, and increased adverse clinical outcomes, including increased morbidity and mortality.^2–4^

Despite the common occurrence of agitation in Alzheimer dementia, the associated healthcare burden has been challenging to characterize, in part because definitions of agitation in real-world research varied widely until development of the provisional International Psychogeriatric Association (IPA) consensus definition of agitation in cognitive disorders in 2015.^5,6^ The IPA definition of agitation requires that patients meet criteria for cognitive impairment or dementia syndrome and exhibit persistent or recurrent (lasting ≥2 weeks) behaviors associated with emotional distress (within the categories of excessive motor activity, verbal aggression, and physical aggression) that are severe enough to cause excess disability and cannot be solely attributed to other conditions or to the physiological effects of a substance.^5^ The provisional IPA definition of agitation was evaluated as a diagnostic tool and validated in community-dwelling older adults with cognitive impairment.^7^ The 2015 IPA provisional definition enabled greater delineation of agitation as a discrete syndrome, and the diagnostic nomenclature has gained wide recognition.^6^ In 2023, the IPA modified the definition of agitation to accommodate special circumstances not anticipated with the development of the provisional definition, thereby establishing the final definition.^8^ The final IPA definition provides guidance to adjust for circumstances where observation of the persistence and severity of behaviors is not possible (eg, agitation in the emergency department or agitation associated with traumatic brain injury).^8^

Medicare health insurance claims data are considered an informative source for surveillance analyses of dementia given that beneficiaries represent the general population aged 65 years and older.^9^ In the United States, out of 59.9 million individuals enrolled in Medicare in 2018, 52 million were aged 65 years or older.^10,11^ However, at the time of this analysis, there was no *International Classification of Diseases, Ninth Revision, Clinical Modification* (ICD-9-CM) or ICD-10-CM code specific to agitation in Alzheimer dementia. We used a claims-based method to identify and characterize patients with agitation in AD using the 2015 IPA definition of agitation (the study was designed prior to the publication of the 2023 definition).^5,6^ To our knowledge, this is the first real-world study to assess the burden of agitation in AD from claims data using the IPA definition of agitation.

Here, we aim to compare patients with agitation in Alzheimer dementia to those without agitation in Alzheimer dementia with respect to patient characteristics, healthcare resource utilization, and costs to characterize the increased healthcare burden among the agitation in Alzheimer dementia population.

## METHODS

### Data Source

This was a retrospective claims database analysis of beneficiaries enrolled in the Medicare Fee-For-Service (FFS) public health insurance program administered by the Centers for Medicare and Medicaid Services (CMS). People aged 65 years or older, younger than 65 years of age with certain disabilities, and people of all ages with end-stage renal disease are eligible for enrollment in Medicare.

Data were extracted from 100% of the Medicare FFS population through a Research Data Use Agreement with CMS, including patient demographics and enrollment coverage; medical claims relating to dates of service, diagnoses, tests, and procedures for inpatient and outpatient care (Parts A and B of the program); and retail, mail order, and specialty pharmacy claims (Part D) during the study period from July 1, 2009, through December 31, 2016.

### Agitation in Alzheimer Dementia Patient Identification

**Figure 1.**
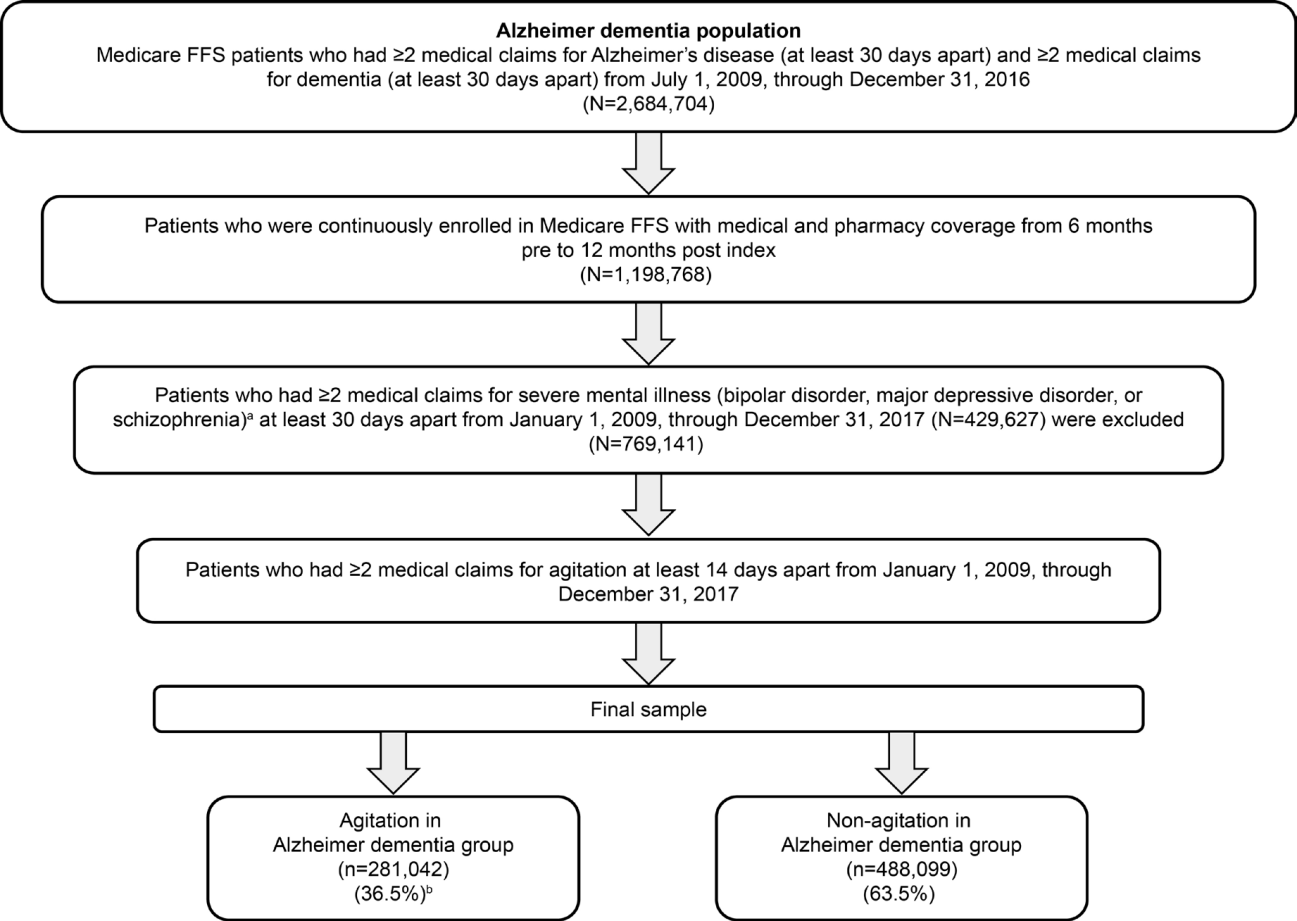
Study Sample Selection Process Based on Provisional IPA Definition Patients had 2 or more medical claims (≥30 days apart) per severe mental illness. Patients can have more than 1 severe mental illness. The percentage represents the estimated prevalence of agitation in Alzheimer dementia as a proportion of patients with AD based on Medicare FFS population. Abbreviations: FFS, Fee-for-Service; IPA, International Psychogeriatric Association.

Identification of patients with agitation in Alzheimer dementia followed an iterative process (**Figure 1**) using readily available claims data. In the first step, strict enrollment criteria and index date rules were applied to confirm a diagnosis of AD, which required 2 or more claims ≥30 days apart in the primary or secondary diagnosis field on the claim during the study identification period from July 1, 2009, through December 31, 2016, for both underlying diagnoses of AD and dementia. The date of the first medical claim for the more recently used qualifying ICD-9-CM or ICD-10-CM diagnosis code (AD or dementia) was used as the index date in this analysis (**Supplemental Table S1**). Thus, if a patient had multiple claims for AD and dementia within the identification period, the service date of the first medical claim for the second of the 2 diagnostic codes (ie, confirming both AD and dementia to ensure that patients had symptomatic dementia associated with AD) was used as the index date. Patients were also required to be continuously enrolled with both medical and pharmacy coverage for 6 months before (baseline period) and 12 months after (follow-up period) the index diagnosis.

To be eligible as having agitation among the identified population with AD, patients were required to have 2 or more claims at least 14 days apart with selected ICD-9-CM/ICD-10-CM codes for agitation (**Supplemental Table S2)**. The agitation diagnosis codes were selected based on behavioral criteria defined in the provisional IPA consensus definition of agitation, which specifies behaviors within the categories of excessive motor activity (eg, R463), verbal aggression (eg, R455), or physical aggression (eg, R456).^6^

Another step involved applying the IPA criteria to assess whether the agitation behaviors were severe enough to produce excess disability, which was defined as including at least 1 of the following: significant impairment in interpersonal relationships, significant impairment in other aspects of social functioning, or significant impairment in the ability to perform or participate in daily living activities. However, we did not use this criterion because of the large number of cases that did not have this information identified in claims, which would have resulted in a drastic reduction in sample size. The final step involved the exclusion of patients identified whose agitation might have been attributable to another psychiatric disorder. As a result, patients with 2 or more medical claims for ICD-9-CM/ICD-10-CM diagnosis codes ≥30 days apart relating to severe psychiatric disorders (bipolar disorder, major depressive disorder, or schizophrenia) were excluded **(Figure 1)**. Patients who did not meet the criteria for agitation comprised the comparator group (without agitation in Alzheimer dementia). Prior to analyzing healthcare resource utilization, an expert panel (1 psychiatrist and 1 neurologist, both MD physicians with research and clinical practice experience in the United States) assessed the face validity of the agitation in Alzheimer dementia algorithm (considering the IPA definition for agitation) and validated it by comparing the demographic and clinical characteristics of the agitation in Alzheimer dementia and non-agitation in Alzheimer dementia groups to confirm that differences were aligned with clinician real-world experience with the agitation in the Alzheimer dementia population.^12^

## Outcome Measures

**Assessment of baseline patient characteristics:** The following characteristics were compared in the agitation and non-agitation groups: baseline demographics (age, sex, race/ethnicity); census region; dual eligibility for Medicare and Medicaid (a proxy measure of low-income status); disability status (determined by original reason for enrollment in Medicare before age 65 due to disability); and 2 measures of clinical severity: CMS–Hierarchical Condition Category (CMS-HCC) risk score; and Charlson Comorbidity Index (CCI) score.

**Healthcare resource utilization and costs during follow-up:** Among the agitation and non-agitation groups, measures of healthcare resource utilization (annualized per 100 members per year) were all-cause and AD-specific hospitalizations and length of stay (LOS); ED and outpatient visits; 30-day readmissions; and number of post-acute care days overall and by setting (skilled nursing facility, home health agency, long-term acute care hospital, inpatient rehabilitation facility, and hospice stays) per patient were estimated.

Total healthcare costs and costs by category (ED, inpatient, outpatient, physician services/tests, durable medical equipment, prescription drug, and post-acute care overall) per patient per year (PPPY) were also compared between the agitation group and the non-agitation group.

**Statistical analysis:** Descriptive statistics were used to compare the agitation group with the non-agitation group for baseline characteristics, healthcare resource utilization, and costs. Categorical variables are shown as frequencies and percentages and continuous variables (including CMS-HCC and CCI scores) as mean (SD) values. All analyses were performed with SAS software, version 9.4 (SAS Institute Inc., Cary, NC, USA).

## RESULTS

Among 2 684 704 patients with AD, 769 141 met criteria for inclusion in this analysis (**Figure 1**). Over half (55.3%) of patients meeting AD criteria were excluded based on requirement for continuous enrollment with both medical and pharmacy coverage 6 months before and 12 months after index diagnosis. Of the remaining AD population, 429 627 (35.8%) were excluded based on co-occurring severe mental illness. After the application of exclusion criteria, 281 042 (36.5%) patients with agitation in Alzheimer dementia and 488 099 (63.5%) patients without agitation in Alzheimer dementia were identified from the AD population.

### Baseline Characteristics

Baseline characteristics for the agitation and non-agitation groups are summarized in **Table 1**. The average age of patients in both groups was 83 years, with a relatively lower proportion aged 80 years or older in the agitation group compared with the non-agitation group (71.3% vs 74.1%, respectively). The majority of patients in the agitation and non-agitation groups were female (69.7% and 71.8%, respectively). More than 80% of patients with Alzheimer dementia, regardless of the presence of agitation, were White (82.8% and 81.2%, respectively). There were similar proportions of patients in each group from other race categories, although the agitation group included slightly lower proportions of Hispanic/Latino (2.7% vs 3.3%, respectively) and Asian (1.6% vs 2.4%) patients than the non-agitation group.

**Table 1.** Demographic and Baseline Characteristics in Alzheimer Dementia Patient Populations With Agitation and Without Agitation

**Baseline Characteristics**	**Agitation in Alzheimer Dementia Group (n = 281 042)**	**Without Agitation in Alzheimer Dementia Group (n = 488 099)**
Age		
Mean (SD), y	83 (8.0)	83 (7.9)
≥80 y, n (%)	200 350 (71.3)	361 485 (74.0)
≥65 to <80 y, n (%)	75 362 (26.8)	119 033 (24.4)
<65 y, n (%)	5330 (1.9)	7581 (1.6)
Female, n (%)	196 003 (69.7)	350 262 (71.8)
Race/ethnicity, n (%)		
White	232 768 (82.8)	396 396 (81.2)
Black	32 031 (11.4)	55 882 (11.4)
Hispanic/Latino	7706 (2.7)	16 162 (3.3)
Asian	4459 (1.6)	11 516 (2.4)
North American Native	936 (0.3)	1606 (0.3)
Unknown	3142 (1.1)	6537 (1.3)
Region, n (%)		
South	117 192 (41.7)	204 554 (41.9)
Midwest	65 303 (23.2)	114 211 (23.4)
Northeast	61 144 (21.8)	95 987 (19.7)
West	37 043 (13.2)	72 180 (14.8)
Unknown	360 (0.1)	1167 (0.2)
Original Medicare entitlement, n (%)		
Aged ≥65 y	275 712 (98.1)	480 518 (98.4)
Disability	29 377 (10.5)	45 920 (9.4)
ESRD	94 (<0.1)	206 (<0.1)
Disability and ESRD	135 (<0.1)	307 (0.1)
Dual eligibility for Medicare and Medicaid, n (%)	126 910 (45.2)	203 657 (41.7)
CMS-HCC risk score, mean (SD)	1.15 (1.52)	1.06 (1.42)
CCI score, mean (SD)	2.50 (2.20)	2.57 (2.30)

Abbreviations: CCI, Charlson Comorbidity Index CMS-HCC, Hierarchical Condition Category; ESRD, end-stage renal disease.

Geographically, most patients originated from the US South in the agitation and non-agitation groups (41.7% and 41.9%, respectively), followed by the Midwest (23.2% and 23.4%), and Northeast (21.8% and 19.7%).

Reaching age 65 was the predominant reason for Medicare entitlement in the agitation (89.5%) and non-agitation (90.5%) groups. The proportion of patients with a disability was slightly higher in the agitation group (10.5%) compared with the non-agitation group (9.4%). In the agitation group, 45.2% of patients qualified for dual-eligibility Medicare and Medicaid status compared with 41.7% in the non-agitation group.

CMS-HCC risk scores are used to estimate future healthcare costs of Medicare beneficiaries. Mean (SD) CMS-HCC risk scores exceeded 1 in both groups but were higher in the agitation group (1.15 [1.52]) than in the non-agitation group (1.06 [1.42]), indicating that the agitation group had a higher risk of future healthcare costs. CCI provides a weighted score of a person’s disease severity that accounts for both the number and severity level of comorbid conditions as they relate to risk of mortality. Mean CCI score was slightly lower in the agitation group compared with the non-agitation group (2.5 vs 2.57), suggesting a slightly higher comorbidity and burden of illness in the non-agitation group. Among prespecified comorbidities and infections of interest, there were no clear trends between the agitation and non-agitation groups, although rates were slightly higher in the non-agitation group, reflecting the observed difference in mean CCI score (**Supplemental Table S3**).

### Healthcare Resource Utilization and Costs

Healthcare resource utilization is summarized for the agitation in Alzheimer dementia and non-agitation in Alzheimer dementia groups in **Table 2**. Hospitalizations, both all cause and AD specific, were higher in the agitation group compared with the non-agitation group. Post-acute care LOS overall was longer in the agitation group (87.7 days) compared with the non-agitation group (80.4 days). In addition, hospital readmission rates were higher (21.8% vs 19.3%, respectively), and outpatient and emergency visits were more frequent in the agitation group. All-cause hospitalizations were 89 per 100 patients per year (P100PPY) in the agitation group compared with 81 P100PPY in the non-agitation group, or 9.9% higher. Alzheimer dementia-specific hospitalization rates were also higher in the agitation group. Emergency department visits were also 20% higher in the agitation group with 115 P100PPY vs 96 in the non-agitation group. Patients in the agitation group incurred 7.3% higher total healthcare costs PPPY than patients without agitation. Prescription drug use and post-acute care (particularly skilled nursing facility stays) were higher among patients with agitation in Alzheimer dementia compared with those without agitation in Alzheimer dementia, and inpatient costs were higher in the agitation population compared with the non-agitation population (**Table 2**).

**Table 2.** Healthcare Resource Utilization

**Healthcare Resource Utilization After Treatment Index (Annualized Per 100 Members per Year)**	**Total Medicare FFS Population**	
	**Agitation in Alzheimer Dementia Group^a,b^ (n = 281 042)**	**Without Agitation in Alzheimer Dementia Group^b^ (n = 488 099)**
Hospitalization (all cause), mean (SD)	89 (122)	81 (118)
LOS, mean (SD) per patient, days	5.97 (11.89)	4.18 (8.21)
LOS, mean (SD) per stay, days	8.96 (10.76)	8.69 (9.92)
Hospitalization (AD specific), mean (SD)	4 (20)	1 (12)
LOS, mean (SD) per patient, days	0.24 (2.71)	0.06 (1.06)
LOS, mean (SD) per stay, days	7.11 (13.18)	4.85 (8.02)
Emergency department, mean (SD)	115 (179)	96 (158)
Outpatient, mean (SD)	1599 (1129)	1476 (1083)
30-day readmission, unadjusted rate (%)	21.8	19.3
Post-acute care LOS, days, mean (SD) per patient		
Overall	87.68 (116.67)	80.38 (114.40)
Skilled nursing facility	33.10 (75.88)	24.07 (64.73)
Home health agency	39.35 (81.23)	40.15 (82.08)
Long-term acute care hospital	0.32 (4.77)	0.28 (3.98)
Inpatient rehabilitation facility	0.26 (2.19)	0.35 (2.51)
Hospice	14.65 (57.35)	15.52 (60.70)
Post-acute care LOS, mean (SD) per stay, days		
Overall	142.65 (119.61)	139.29 (120.31)
Skilled nursing facility	93.52 (103.04)	84.19 (97.95)
Home health agency	107.63 (103.43)	105.09 (103.98)
Long-term acute care hospital	32.29 (35.90)	31.03 (27.99)
Inpatient rehabilitation facility	13.37 (8.50)	13.26 (8.14)
Hospice	152.45 (114.94)	163.42 (120.94)
Proportion of patients requiring post-acute care, n (%)		
Overall	168 747 (60.0)	273 137 (56.0)
Skilled nursing facility	99 472 (35.4)	139 566 (28.6)
Home health agency	102 752 (36.6)	186 488 (38.2)
Long-term acute care hospital	2755 (1.0)	4458 (0.9)
Inpatient rehabilitation facility	5427 (1.9)	12 901 (2.6)
Hospice	27 015 (9.6)	46 356 (9.5)

^a^Population identified through medical claims data (≥2 diagnosis codes for agitation ≥14 days apart from January 1, 2009–December 31, 2017).

^b^Excluded patients who had 2 or more medical claims for severe mental illness (bipolar disorder, major depressive disorder, or schizophrenia) at least 30 days apart from January 1, 2009, through December 31, 2017. Patients had to have 2 or more medical claims (≥30 days apart) per severe mental illness.

Abbreviation: LOS, length of stay.

**Figure 2.**
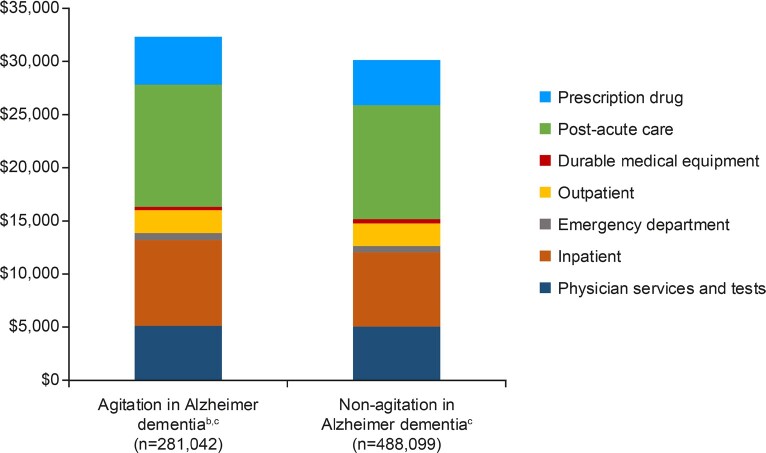
Total Direct Healthcare Costs Post Treatment Index in Patients With and Without Agitation Post-acute care includes long-term acute care hospitalization, inpatient rehabilitation facility, skilled nursing facility, and home health agency. ^a^Per-patient per-year after treatment index. ^b^Population identified through medical claims data (≥2 diagnosis codes for agitation ≥14 days apart from July 1, 2009–December 31, 2017). ^c^Excluded patients who had 2 or more medical claims for severe mental illness (bipolar disorder, major depressive disorder, or schizophrenia) at least 30 days apart from January 1, 2009, through December 31, 2017. Patients had to have 2 or more medical claims (≥30 days apart) per severe mental illness.

The mean total direct post-index healthcare cost in patients with and without agitation were $32 322 and $30 121 PPPY, respectively (**Figure 2**). Most medical expenditures (mean cost PPPY) were numerically higher among the agitation group compared with the non-agitation group, with the largest apparent differences in inpatient costs and post-acute care (physician services and tests: $5149 and $5080, inpatient costs: $8062 and $6970, emergency department (ED) costs: $652 and $581, outpatient services: $2152 and $2133, post-acute care: $11 487 and $10 700; and prescription drug costs: $4496 and $4237, respectively). Durable medical equipment expenditures (mean cost PPPY) were numerically lower in the agitation group compared with the non-agitation group ($324 and $420).

## DISCUSSION

To our knowledge, this is the first study to develop an algorithm using the provisional IPA consensus definition to identify patients with agitation in Alzheimer dementia in claims data from the US Medicare population. The algorithm identified a cohort of patients with agitation in Alzheimer dementia who were descriptively compared with patients without agitation with respect to baseline characteristics, healthcare resource utilization, and costs. Overall, the prevalence of agitation in Alzheimer dementia was 36.5%. When characteristics of the identified agitation in Alzheimer dementia group were compared with the non-agitation group, age 65 to <80 years (26.8% vs 24.4%, respectively), male sex, and dual eligibility for Medicare and Medicaid (as a proxy for lower socioeconomic status) were more common among patients with agitation. The trend toward lower socioeconomic status in those with agitation is an important finding, given that previous studies have found an association between dementia risk and socioeconomic status.^13^ Healthcare costs were consistently higher in the agitation group compared with the non-agitation group. A greater number of hospitalizations and ED visits in the agitation in Alzheimer dementia population may have contributed to higher healthcare resource utilization and costs. However, additional analyses detailing the precise factors driving differences in costs are warranted.

Given the high prevalence of dementia, the resource utilization and economic burden associated with agitation in patients with Alzheimer dementia is substantial and is aligned with other published research.^14,15^ Grossberg et al found that agitation in AD was associated with increased healthcare costs based on US electronic medical record and administrative claims data, with agitation-related incremental costs $4287 PPPY higher in the agitation cohort compared with the non-agitation cohort.^16^ A UK-based study established a significant economic burden of agitation exceeding that of cognitive impairment alone in patients with AD and highlighting the need for effective interventions to reduce agitation and associated costs.^15^ Our results are also aligned with findings of Aigbogun et al, who analyzed an AD cohort with behavioral disturbance (BD) from a US claims database; their study was conducted in a similar time frame to that of this study (2012-2015 vs 2009-2016 in our study), and they reported greater healthcare utilization and costs associated with BD.^14^ Although BD is a broader, less precisely defined parameter than the IPA consensus definition of agitation, the work of Aigbogun et al may still serve as a comparative benchmark. Another recent US-based retrospective analysis (of the National Alzheimer’s Coordinating Center Uniform Data Set) reported that a 20% higher risk of institutionalization associated with agitation in patients with AD translated to a total incremental cost of $4.3 billion, equivalent to more than $50 000 per patient.^17^ A separate retrospective analysis of physician-reported patient data from a point-in-time survey compared agitated and non-agitated US patients with dementia.^18^ Agitation in dementia was found to be linked to increased healthcare resource utilization and healthcare costs, as evidenced by the greater number of professional caregivers, institutionalizations, hospitalizations in a psychiatric ward, treatments received, and consultations compared with non-agitated patients with Alzheimer dementia.^18^

We found a numerically higher mean CCI score in the non-agitation group at baseline, which is indicative of a higher comorbidity burden compared with the agitation group. It may be that patients with multiple chronic conditions are too frail to exhibit agitation, but further research is needed to investigate this hypothesis.

Although the IPA definition was applied as accurately as possible within the claims, there are limitations to implementing the IPA definition using only administrative claims data. Underestimation of agitation in Alzheimer dementia prevalence due to miscoding of AD-related dementias in claims databases has been reported,^19,20^ although linkage between Medicare and medical chart data showed that approximately 85% of patients with a clinical diagnosis of Alzheimer dementia can be identified using claims data.^20^ New codes available since October 2022 may help to better identify this patient population; however, despite improvements in coding, all criteria from the IPA definition regarding agitation should be considered in identifying patients with agitation. Importantly, claims data may not capture all relevant behaviors to align with the persistence of symptoms and the emotional distress components of the IPA definition of agitation. The exclusion of patients with 2 or more claims for severe mental illness at least 30 days apart in the study period may limit the generalizability of findings, as patients would be excluded if their agitation symptoms could be attributed to a comorbid severe mental illness.

The present study has several additional limitations. While the large sample sizes used in this study would render all differences to be statistically significant, the analysis was not designed for formal statistical comparisons; thus, all comparisons based on descriptive statistics should be considered hypotheses. Enrollment in Medicare before age 65 was used as a proxy for disability status, based on the CMS definition of disability; therefore, overall disability status in the population may be undercounted, as some patients may have developed a disability after initial Medicare enrollment at age 65. Other limitations of the study are inherent to claims-based analyses, such as incomplete or missing data and applying caution when considering the generalizability of these findings from the Medicare FFS to the overall US population. However, it is noteworthy that the Medicare population is drawn from a large proportion of the US population aged 65 and older, which represents a large percentage of patients with AD.

## CONCLUSIONS

Greater healthcare resource utilization and costs associated with patients with agitation in Alzheimer dementia underscore the important need for interventions to address agitation, which in time may positively impact health outcomes and associated Medicare costs. The methodology developed in this analysis will be vital in future claims-based studies to help connect data based on older ICD codes to newly developed codes and to comprehensively understand how pharmacological and non-pharmacological interventions impact healthcare resource utilization and costs in patients with Alzheimer dementia.^10,11^

### Disclosures

C.T. and Z.P. report no conflicts of interest. D.B. and S.H. are employees of Otsuka Pharmaceuticals. This study was funded by Avanir Pharmaceuticals and conducted independently by Inovalon.

## Supplementary Material

Online Supplementary Material
