# Supplementary material for: Healthcare Resource Utilization Among Patients With Agitation in Alzheimer Dementia
Source: J Health Econ Outcomes Res. 2024 Oct 29;11(2):118–24. doi: 10.36469/001c.124455 (PMC11539929; doi:10.36469/001c.124455)
Supplement: Online Supplementary Material [file jheor_2024_11_2_124455_251367.pdf]

Journal of Health Economics and Outcomes Research

Online Supplementary Material

Healthcare Resource Utilization Among Patients With Agitation in Alzheimer Dementia. *JHEOR*. 2024;11(2):118-124. doi:10.36469/jheor.2024.124455

Table S1: Alzheimer's Disease and Dementia ICD-9/10-CM Codes

Table S2: Patients With Agitation in Alzheimer Dementia by ICD-9/10-CM Code

Table S3: Prespecified Comorbidities and Infections at Baseline

This supplementary material has been provided by the authors to give readers additional information about their work.

This is an open-access article distributed under the terms of the Creative Commons Attribution 4.0 International License (CCBY-4.0). View this license's legal deed at http://creativecommons.org/licenses/by/4.0 and legal code at http://creativecommons.org/licenses/by/4.0/legalcode for more information.

Table S1. Alzheimer's Disease and Dementia ICD-9/10-CM Codes

Code Type	Code Value	Code Description
Alzheimer's disease		
ICD-10-CM	G300	Alzheimer's Disease with early onset
ICD-10-CM	G301	Alzheimer's Disease with late onset
ICD-10-CM	G308	Other Alzheimer's Disease
ICD-10-CM	G309	Alzheimer's Disease, unspecified
ICD-9-CM	3310	Alzheimer's Disease
Dementia		
ICD-10-CM	F0280	Dementia in other diseases classified elsewhere without behavioral disturbance
ICD-10-CM	F0281	Dementia in other diseases classified elsewhere with behavioral disturbance
ICD-9-CM	29411	Dementia in conditions classified elsewhere with behavioral disturbance
ICD-10-CM	F0390	Unspecified dementia without behavioral disturbance
ICD-10-CM	F0391	Unspecified dementia with behavioral disturbance
ICD-9-CM	29410	Dementia in conditions classified elsewhere without behavioral disturbance
ICD-9-CM	2900	Senile dementia, uncomplicated
ICD-9-CM	29010	Presenile dementia, uncomplicated
ICD-9-CM	29012	Presenile dementia with delusional features
ICD-9-CM	29013	Presenile dementia with depressive features
ICD-9-CM	29020	Senile dementia with delusional features
ICD-9-CM	29021	Senile dementia with depressive features
ICD-9-CM	29420	Dementia, unspecified, without behavioral disturbance
ICD-9-CM	29421	Dementia, unspecified, with behavioral disturbance

Abbreviation: ICD-9/10-CM, *International Classification of Diseases, Ninth/Tenth Revision, Clinical Modification.*

Table S2. Patients With Agitation in Alzheimer Dementia by ICD-9/10-CM Code

Patients With Agitation Identified by ICD-9/10-CM Codes, n (%)			Agitation in Alzheimer Dementia Population[a] (n = 281 042)
**Code Type**	**Code Value**	**Code Description**	
		Psychosis condition - severe mental illness (bipolar disorder, major depressive disorder, and schizophrenia)	0 (0.0)
ICD-9-CM	29411	Dementia in conditions classified elsewhere with behavioral disturbance	209 324 (74.5)
ICD-9-CM	29421	Dementia, unspecified, with behavioral disturbance	86 297 (30.7)
ICD-9-CM	79922	Irritability	2531 (0.9)
ICD-10-CM	F0281	Dementia in other diseases classified elsewhere with behavioral disturbance	75 681 (26.9)
ICD-10-CM	F0391	Unspecified dementia with behavioral disturbance	60 135 (21.4)
ICD-10-CM	F423	Hoarding disorder	27 (0.0)
ICD-10-CM	R451	Restlessness and agitation	22 376 (8.0)
ICD-10-CM	R454	Irritability and anger	1279 (0.5)
ICD-10-CM	R455	Hostility	137 (0.0)
ICD-10-CM	R456	Violent behavior	961 (0.3)
ICD-10-CM	R4587	Impulsiveness	315 (0.1)
ICD-10-CM	R463	Overactivity	<11 (N/A)
ICD-9-CM	V4031	Wandering in diseases classified elsewhere	6543 (2.3)
ICD-10-CM	Z9183	Wandering in diseases classified elsewhere	4090 (1.5)

[a]Population identified through medical claims data (≥2 diagnosis code for agitation ≥14 days apart from July 1, 2009, through December 31, 2016). Excluded patients who had ≥2 medical claims for severe mental illness (bipolar disorder, major depressive disorder, or schizophrenia) ≥30 days apart from January 1, 2009, through December 31, 2017. Patients had to have ≥2 medical claims (≥30 days apart) per severe mental illness.
Abbreviation: ICD-9/10-CM, *International Classification of Diseases, Ninth/Tenth Revision, Clinical Modification.*

Table S3. Prespecified Comorbidities and Infections at Baseline

Patients With Prespecified Comorbidities or Infections, n (%)	Agitation in Alzheimer Dementia Group (n = 281 042)	Without Agitation in Alzheimer Dementia Group (n = 488 099)
Hypertension	208 271 (74.1)	367 371 (75.3)
Dyslipidemia	130 382 (46.4)	237 121 (48.6)
Peripheral vascular disease	122 729 (43.7)	217 567 (44.6)
Urinary tract infection	87 488 (31.1)	141 871 (29.1)
Osteoarthritis	84 040 (29.9)	149 119 (30.6)
Diabetes	79 927 (28.4)	145 126 (29.7)
Ischemic heart disease	76 656 (27.3)	142 511 (29.2)
Heart failure	50 402 (17.9)	99 288 (20.3)
Chronic obstructive pulmonary disease	47 995 (17.1)	91 481 (18.7)
Cerebrovascular disease	43 156 (15.4)	80 973 (16.6)
Osteoporosis	40 620 (14.5)	75 165 (15.4)
Atherosclerosis	37 338 (13.3)	64 258 (13.2)
Acute respiratory infection	29 656 (10.6)	56 628 (11.6)
Pneumonia	27 409 (9.8)	52 403 (10.7)
Chronic ulcer of the skin	22 185 (7.9)	42 079 (8.6)
Epilepsy	18 206 (6.5)	30 528 (6.3)
Venous thromboembolism	13 272 (4.7)	24 289 (5.0)
Gastric, duodenal, peptic, or gastrojejunal ulcer	12 717 (4.5)	25 183 (5.2)
Mild cognitive impairment	8374 (3.0)	16 833 (3.4)
Rheumatoid arthritis	5981 (2.1)	12 305 (2.5)
